# Multiple *trans* QTL and one *cis*-regulatory deletion are associated with the differential expression of cone opsins in African cichlids

**DOI:** 10.1186/s12864-018-5328-z

**Published:** 2018-12-18

**Authors:** Sri Pratima Nandamuri, Matthew A. Conte, Karen L. Carleton

**Affiliations:** Department of Biology, University of Maryland, 1210 Biology / Psychology Bldg #144, College Park, MD 20742 USA

**Keywords:** QTL, Cichlid, Opsin, *Cis*-regulation, *SWS1*

## Abstract

**Background:**

Dissecting the genetic basis of phenotypic diversity is one of the fundamental goals in evolutionary biology. Despite growing evidence for gene expression divergence being responsible for the evolution of complex traits, knowledge about the proximate genetic causes underlying these traits is still limited. African cichlids have diverse visual systems, with different species expressing different combinations of seven cone opsin genes. Using opsin expression variation in African cichlids as a model for gene expression evolution, this study aims to investigate the genetic architecture of opsin expression divergence in this group.

**Results:**

Results from a genome-wide linkage mapping on the F_2_ progeny of an intergeneric cross, between two species with differential opsin expression show that opsins in Lake Malawi cichlids are controlled by multiple quantitative trait loci (QTLs). Most of these QTLs are located in *trans* to the opsins except for one *cis*-QTL for *SWS1* on LG17. A closer look at this major QTL revealed the presence of a 691 bp deletion in the promoter of the *SWS1* opsin (located 751 bp upstream of the start site) that is associated with a decrease in its expression. Phylogenetic footprinting indicates that the region spanning the deletion harbors a microRNA *miR-729* and a conserved non-coding element (CNE) that also occurs in zebrafish and other teleosts. This suggests that the deletion might contain ancestrally preserved regulators that have been tuned for *SWS1* gene expression in Lake Malawi. While this deletion is not common, it does occur in several other species within the lake.

**Conclusions:**

Differential expression of cichlid opsins is associated with multiple overlapping QTL, with all but one in *trans* to the opsins they regulate. The one *cis*-acting factor is a deletion in the promoter of the *SWS1* opsin, suggesting that ancestral polymorphic deletions may contribute to cichlid’s visual diversity. In addition to expanding our understanding of the molecular landscape of opsin expression in African cichlids, this study sheds light on the molecular mechanisms underlying phenotypic variation in natural populations.

**Electronic supplementary material:**

The online version of this article (10.1186/s12864-018-5328-z) contains supplementary material, which is available to authorized users.

## Background

Deciphering the genetic mechanisms underlying phenotypic diversity has long been at the forefront of evolutionary biology research. Much of what we know about the molecular basis for phenotypic variation comes from studies in classic model systems such as yeast and *Drosophila* [1–5]. This work has, without any doubt, vastly advanced our understanding of trait evolution. We now know that the emergence of novel phenotypes can result from both mutations in coding sequences that alter the structure or function of the proteins, or by mutations in the regulatory sequences that modify the spatial and temporal expression patterns of genes. There have been animated debates in the literature on the importance of coding sequence versus regulatory mutations and the relative contribution of *cis* versus *trans* regulatory mutations in bringing about phenotypic change [6–9]. Although theory predicts that *cis*-regulatory mutations must predominate owing to their highly specific effects and reduced pleiotropy, results from actual studies are often conflicting [10–12]. Adding to the confusion is the fact that some of these studies were performed using inbred wild type and mutant lab raised individuals. Consequently, knowledge about the genetic processes responsible for phenotypic divergence in natural populations has been severely limited. However, recent advances in cost effective sequencing methods allow us to unravel the mechanistic basis for natural adaptation by combining sequence information with traditional quantitative trait loci mapping and candidate gene approaches. Such methods have recently been employed to uncover skeletal appendage patterning in sticklebacks, eye loss in *Astyanax*, feathered feet in pigeons and coat coloration in beach mice, among others [13–17]. Yet a coherent understanding of the relative roles of *cis*- versus *trans-* regulatory changes requires more studies representing a broader set of traits in naturally-occurring populations.

Vertebrate opsins include four main cone opsin families - *SWS1*, *SWS2*, *RH2* and *LWS* as well as the *RH1* family found in rods [18–21]. Experimental work in a variety of model systems has identified key factors governing photoreceptor differentiation and opsin expression [22, 23]. These studies have identified that opsins are controlled both by *cis*-factors and *trans*-acting elements. For example, studies in humans have shown that opsin genes are controlled by *cis*-enhancers located upstream of the genes [24]. Similarly, work in mice has shown that *Thyroid Hormone Receptor Beta* (*THRβ)* transcription factor specifies photoreceptors to a M-cone (*LWS* family) fate [25]. Nonetheless, these studies fail to present a complete understanding of the molecular mechanisms underlying differential opsin expression, simply because model organisms such as mice and humans do not express representatives from all cone families. Owing to gene duplication events, fish typically have a more complex set of cones that are members of the four prototypical vertebrate cone classes [26]. This provides ample opportunity to study genetic factors underlying the specification of opsins that are usually absent in many other vertebrates, such as *SWS1*. In fact, separate studies in zebrafish and trout have identified that *SWS1* opsin is downregulated by *THRβ* and upregulated by *TBX2B* respectively [27, 28]. However, no studies have yet been done to examine variable *SWS1* opsin expression between closely related species in either trout or zebrafish and mechanisms driving the differential expression of *SWS1* opsin largely remain unknown.

African cichlids are a particularly promising system to study the genetic basis of phenotypic diversity [29–31]. This highly speciose group of freshwater fish inhabit rift lakes throughout Africa, most notably in the three great lakes- Malawi, Victoria and Tanganyika [32]. Incredible bursts of parallel adaptive radiations have occurred in each of these lakes, making cichlids a classical example of explosive speciation. [33–35]. Just in Lake Malawi, more than 500 species have arisen in the last few million years following multiple repeated colonization and hybridization events [36, 37]. Cichlids are amenable for studying the genetics of adaptive traits for three main reasons. First, different species and even genera of cichlids can interbreed to produce fertile hybrids [38–40]. This makes mapping of traits for intraspecific and even interspecific comparisons possible. Second, owing to their recent divergence, key polymorphisms are likely to be shared between species [41]. Besides enabling us to find causative mutations, inferences about the evolutionary history of the trait can be easily drawn. Third, well-developed genomic resources are available for cichlids, with complete genomes for the riverine ancestor Tilapia and representative species from each of the three great lakes [42].

Within Lake Malawi, cichlids exhibit incredible diversity for a variety of anatomical, physiological and behavioral traits [43–46]. Closely related species often occupy different habitats, have varied coloration patterns, display numerous feeding strategies and breeding behaviors. For this study, we focus on the diversity of their visual systems, particularly in terms of the genes responsible for color discrimination, the cone opsins. All African cichlid species have seven cone opsin genes in their genomes, each maximally sensitive to a different part of the light spectrum [47]. These include three single cone opsins- *SWS1*(ultraviolet-sensitive), *SWS2B* (violet-sensitive), *SWS2A* (blue-sensitive), and four double cone opsins- *RH2B* (blue-green-sensitive), *RH2A-alpha* and *RH2A*-*beta* (green-sensitive), and *LWS* (red-sensitive). While the *SWS1* opsin in cichlids is located on LG17 (linkage group 17), the remaining opsins form two tandem arrays (*SWS2A-SWS2B-LWS* and *RH2B-RH2Aα-RH2Aβ*) separated by 30 Mb on LG5 [48].

In Lake Malawi cichlids, large variation in visual sensitivities both within and between species has been shown to be mediated not by coding sequence changes but by alterations in the expression of the cone opsin genes [49–51]. As adults, species from Lake Malawi express different subsets of the seven genes, leading to dramatic differences in their visual sensitivities [39, 52]. Besides being important for foraging, predator avoidance and habitat- preference, vision is key for cichlid fitness playing a major role in mate choice and courtship [53–57]. In most species, the males are colorful while females are drab and brown. During breeding season, colorful dominant males display aggressive mating behaviors and get visually selected by choosy females to spawn the next generation [58, 59]. Sister taxa in cichlids often differ in visual sensitivities but not in morphology or behavior. Therefore, it has been hypothesized that the differences in visual capabilities due to variation in opsin gene expression may contribute significantly to the rapid speciation within this group [60, 61].

A genome-wide mapping study using hybrids from an intergeneric cross between *Tramitochromis intermedius* and *Aulonocara baenschi* (TA cross) was previously carried out in our lab to identify genomic regions associated with the differential expression of opsins [62]. Results from that study identified 11 QTL for 5 cone opsins segregating in the cross- 2 loci each for single cone opsins (S*WS2A* and *SWS2B*- LG5, LG23) and 7 loci for double cone opsins (*RH2B*, *RH2A*, *LWS*- LG1, LG5, LG10, LG15 and LG23) Here, *RH2Aα* and *RH2Aβ* are more than 99% identical and thus have been merged together as *RH2A*. Most of these QTLs are positioned on chromosomes that do not contain the opsin genes they are linked to, indicating that the cone opsins on LG5 are not coordinated by *cis*-regulatory regions adjacent to the opsin, but are regulated by *trans-*loci located elsewhere in the genome. Even the QTLs on LG5 are located distantly from the opsin genes, separated by ~ 720 kb, and conservatively classified to be functioning in *trans*. More importantly, since neither of the parental species from the cross had *SWS1* expression, the basis of *SWS1* differential expression could not be determined.

To find genetic loci controlling the differential expression of *SWS1* opsin, the current study relies on a new hybrid cross between two Lake Malawi species- *Aulonocara baenschi* (negligible *SWS1* expression) and *Metriaclima mbenji* (high *SWS1* expression, MA cross) [63]. In cichlids, the *SWS1* gene is a single copy opsin on LG17 separated from the rest of the 6-opsin cluster [48]. Previously, we found an association between *SWS1* expression and a micro-satellite marker located in the third intron of the opsin gene, suggesting that differential expression of *SWS1* opsin could be governed by *cis*-regulation [63]. However, this association was tested with just one marker and only in a subset of the F_2_.

Here, we use restriction-site associated DNA sequencing (RAD-Seq) and QTL mapping with markers across the whole genome, using F_2_ progeny from the same cross to get a more complete picture of the genetic mechanisms governing *SWS1* expression. We find three QTLs associated with the differential expression of *SWS1*- two in *trans* on LG14 and LG20, and one located in *cis* to the *SWS1* opsin gene on LG17, indicating that the modulation of *SWS1* opsin expression evolved through changes in both *cis*-regulatory sequences as well as *trans*-acting factors. Also, we identify additional loci whose segregation is associated with the differential expression of the other opsins (*SWS2B*, *RH2B*, *RH2A* and *LWS*). In addition to presenting a more detailed picture of the genetic architecture of opsin expression in cichlids, this study will lead to a more coherent understanding of the molecular mechanisms contributing to adaptive phenotypic diversity.

## Results

### Opsin expression variation in the F2 hybrids

As described in Nandamuri et al. [63], opsin expression measured by quantitative PCR varied considerably among the 157 F_2_ hybrids in the MA cross. Most opsins showed a dominant pattern of inheritance in the F_2_ hybrids, with expression of *SWS1*, *SWS2B* and *RH2B* leaning towards levels observed in *A. baenschii* F_0_, while that of *RH2A* was closer to levels in *M. mbenji* F_0_. Consistent with expression in both the F_0_ parents, the F_2_ hybrids showed negligible levels of *SWS2A* expression. Expression of *LWS* opsin in the double cones was transgressive with F_2_ hybrids showing higher expression than either of the parents of the cross. Since expression of each opsin in the cross is characterized as a fraction of total expression in single cones (*SWS1*, *SWS2B*, *SWS2A*) and double cones (*RH2B*, *RH2A*, *LWS*) respectively, and since most F_2_ hybrids show negligible *SWS2A* expression, *SWS1*and *SWS2B* opsins become inversely correlated.

### RAD-Seq genotyping

After removal of reads with ambiguous barcodes, Illumina sequencing of the two RAD-Seq libraries yielded a total of ~ 185 million reads for the F_2_ hybrids. Around 97% of these reads (~ 178 million) passed the quality filters and were retained for subsequent processing. This represented a total of ~ 66,500 SbfI restriction sites in the cichlid genome, corresponding to an average read depth of 17x for each F_2_ individual. Similarly, after filtering and barcode processing, the average read coverage in the *M. mbenji* F_0_ and *A. baenschii* F_0_ was 16x (~ 1 million reads) and 70x (~ 4.6 million reads) respectively. The low coverage in the *M. mbenji* F_0_ was due to the low-quality DNA. The RAD-Seq processing statistics for the F_2_ and the F_0_ individuals are provided in Additional file 1. In total, Stacks software identified 2370 RAD-Seq SNPs (single-nucleotide polymorphisms) following stringent cutoffs. After filtering to identify differentially fixed SNPs in the F_0_, anchoring to a unique location in the *M. zebra* genome and adherence to Hardy-Weinberg equilibrium, we retained a conservative set of 1217 SNPs for linkage map and QTL analysis. The FASTA sequences of the 1217 RAD markers are provided in Additional file 2. The total fraction of missing genotypes across all 1217 SNP markers and 157 F_2_ individuals was 4.5% while the frequency of each genotypic class was 26.1% homozygous for *M. mbenji* alleles (MM), 25.4% homozygous for *A. baenschi* alleles (AA) and 48.5% heterozygous (MA).

### Linkage mapping and anchoring to *M. zebra* genome

A high-density linkage map comprising 22 linkage groups spanning 1558.1 cM was assembled (Additional file 3: Figure S1). The average intermarker distance was 1.3 cM and the maximum distance between consecutive markers was 20.9 cM. Previous karyotyping work has shown that a haploid cichlid genome is comprised of 22 chromosomes indicating that the linkage map obtained in this study is complete, with one linkage group per chromosome [48]. Also, the length of the map is comparable to previously published linkage maps constructed from other cichlid crosses with closely related species [45, 62, 64]. The linkage map anchored 662,849,923 bp, representing 69.3% of the *M. zebra* genome assembly. The linkage map as well as the phenotype and genotype information used for QTL analysis is provided in Additional file 4.

### QTL for single cone opsins

**Fig. 1:**
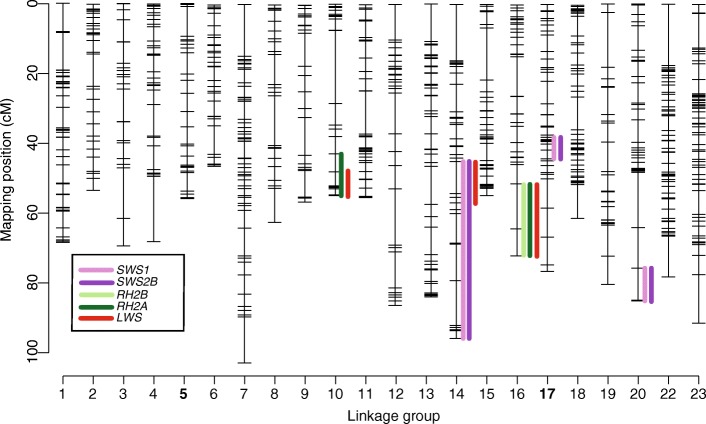
Genome wide distribution of cone opsin QTL. Quantitative trait loci (QTL) mapping detected 12 intervals on 5 LGs that segregated for the differential expression of cone opsins between the two F_0_ species. In the single cones, *SWS1* and *SWS2B* have 3 co-localizing QTLs on LG14, LG17 and LG20. While *RH2B* in the double cones has only 1 QTL on LG16, *RH2A* and *LWS* share 2 QTL on LG10 and LG16. Single cones and double cones share 1 QTL on LG14. Each cone opsin QTL is indicated by a separate color depicted in the color legend. The cone opsins in cichlids are present on two chromosomes- LG5 (*SWS2B*, *SWS2A*, *LWS*, *RH2B*, *RH2Aα*, *RH2Aβ*) and LG17 (*SWS1*). The black lines represent RAD loci

Using quantitative trait locus mapping, we detected a total of nine significant QTL for single cone opsin expression- three for *SWS1* (LG14, LG20 and LG17*)*, three for *SWS2B* (LG14, LG20 and LG17) and three for *SWS2A* (all on LG23) (Fig. 1)*.* However, the three *SWS2A* QTL were likely an artifact of the multiple scan for two reasons: 1) the three loci on LG23 are in close proximity to each other and not separated by at least one genotyped marker. 2) the significant signal is driven by just 10 (of the 157 F_2_) individuals showing less than 2% *SWS2A* expression. Therefore, the *SWS2A* QTL were removed from subsequent analysis. Due to the nature of normalization in the single cones and the absence of *SWS2A* expression in most F_2_ hybrids, overlapping QTLs were obtained for both the *SWS1* and *SWS2B* opsins (Fig. 1). The LOD (logarithm of odds) scores, QTL intervals and percent variance in opsin expression (PVE) explained by each of the QTLs are provided in Table 1.

**Table 1:** Summary of QTL for single cone opsin expression in the MA cross

Opsin	LG	Location (cM)	LOD	PVE	95% BI (cM)	95% BI (markers)
*SWS1*	17	39.28	17.97	31.65	38.02–44.21	RAD_33279 to RAD_39835
*SWS1*	20	81	8.58	13.04	75.64–85.07	RAD_22102 to RAD_52696
*SWS1*	14	56.81	6.62	9.77	44.96–95.69	RAD_41335 to RAD_2689
*SWS2B*	17	39.28	17.09	31.3	38.02–44.21	RAD_33279 to RAD_39835
*SWS2B*	20	81	9.04	14.6	75.64–84.86	RAD_22102 to RAD_8560
*SWS2B*	14	92.5	5.68	8.72	44.97–95.69	RAD_41335 to RAD_2689

*LOD* Logarithm of Odds, *PVE* Percentage of variance explained in the F_2_, *BI* 95% Bayes interval

**Fig. 2:**
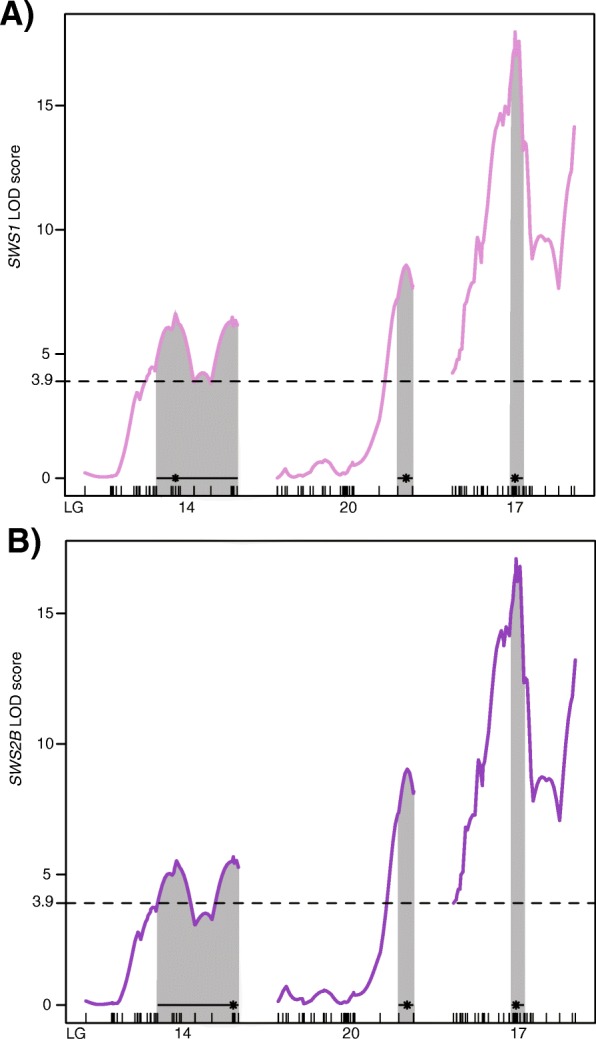
QTL mapping for *SWS1* and *SWS2B* opsins. LOD scores of significant QTL for single cone opsins. Horizontal dotted line indicated genome-wide significance threshold for both opsins at a significance *P* = 0.05. The black line at the bottom of each QTL indicates the 95% Bayes credible interval. The asterisk denotes the location of the most significant marker (highest LOD score) in each QTL. **a** QTL for *SWS1* expression on LG14, LG20 and LG17. The *SWS1* opsin is also located in the Bayes interval of the QTL on LG17, 20 bp from the most significant marker. **b** QTL for *SWS2B* expression on LG14, LG20 and LG17

Genome-wide LOD threshold calculated at a significance of *P* = 0.05 is 3.9 for both the *SWS1* and *SWS2B* opsins. For the *SWS1* opsin, the QTL on LG17, spanning a region of 6.2 cM, is the most significant of the three QTL (LOD = 17.97) (Fig. 2a). Coincidentally, the *SWS1* opsin gene is located within this QTL interval, suggesting that the *SWS1* expression is potentially *cis*-regulated. Additionally, this QTL has the largest effect on *SWS1* expression, accounting for 31.65% of the variance in F_2_ expression. The two QTLs on LG14 (LOD = 6.62) and LG20 (LOD = 8.58) are *trans* QTL explaining 9.8 and 13% variation in *SWS1* expression respectively (Fig. 2a). The *SWS2B* opsin is located on LG5. Therefore, all three QTL found in this study are located in *trans*. Similar to *SWS1*, LG17 (LOD = 17.1) is the largest contributor to *SWS2B* expression, explaining 31.3% of the variance (Fig. 2b). Around 8.7 and 14.6% F_2_ expression variance is explained by the remaining QTL on LG14 (LOD = 5.68) and LG20 (LOD = 9.04) respectively (Fig. 2b).

Effect plots for *SWS1* show that the two QTLs on LG14 and LG17 are additive with higher *SWS1* expression observed in the F_2_ hybrids that are homozygous for the *M. mbenji* alleles (MM>MA>AA) (Additional file 3: Figure S2A). However, the QTL on LG20 shows a dominant pattern of inheritance with both heterozygotes and *A. baenschi* homozygotes showing equal levels of low *SWS1* expression (MM>MA=AA) suggesting that the *Aulonocara* allele is a suppressor of *SWS1* expression (Additional file 3: Figure S2A). Effect plots for *SWS2B* are identical except in the opposite direction (LG14 and LG17: MM<MA<AA; LG20: MM<MA=AA (Additional file 3: Figure S2B)).

### QTL for double cone opsins

**Fig. 3:**
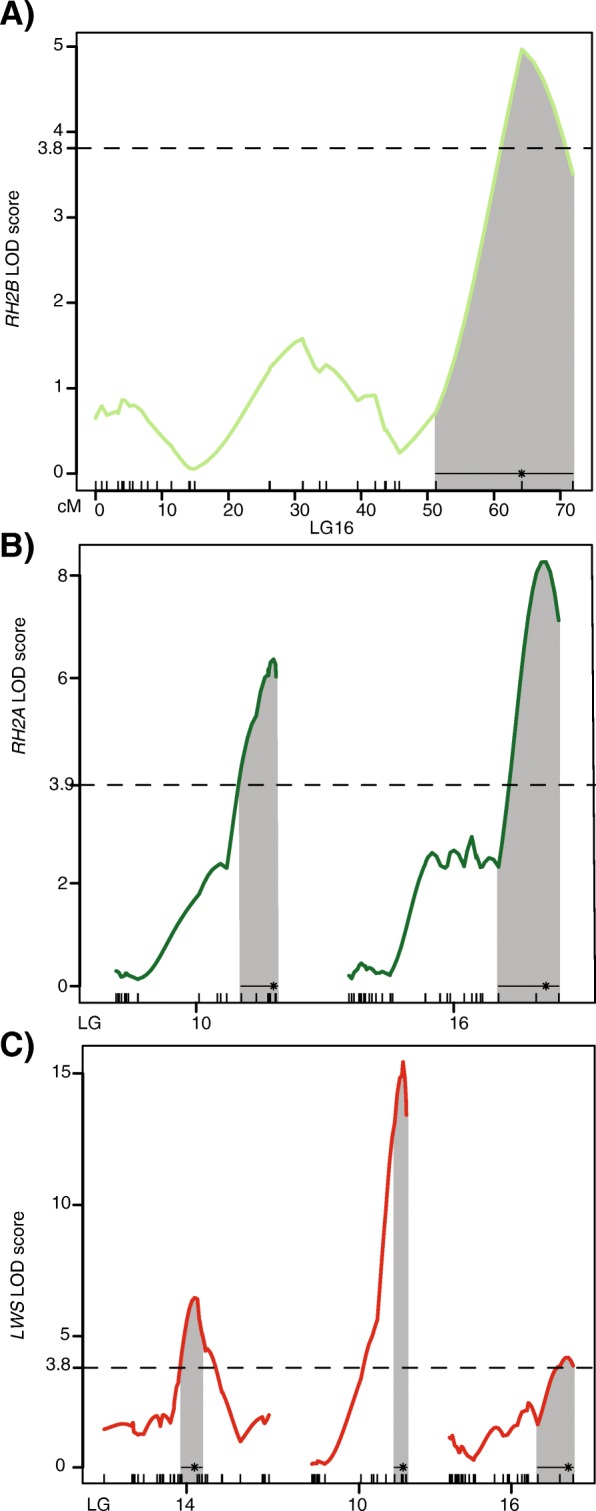
QTL mapping for *RH2B*, *RH2A* and *LWS* opsins. LOD scores of significant QTL for single cone opsins. Horizontal dotted line indicated genome-wide significance threshold for all opsins at a significance *P* = 0.05. The black line at the bottom of each QTL indicates the 95% Bayes credible interval. The asterisk denotes the location of the most significant marker (highest LOD score) in each QTL. **a** QTL for *RH2B* expression on LG16. **b** QTL for *RH2A* expression on LG10 and LG16. **c** QTL for *LWS* expression on LG14, LG10 and LG16

The genome-wide LOD thresholds for *RH2B*, *RH2A* and *LWS* opsins are 3.81, 3.91 and 3.78 respectively. For the double cone opsins, we identified a total of six significant QTL: one for *RH2B* on LG16 (LOD = 4.97); two for *RH2A* on LG10 (LOD = 6.37) and LG16 (LOD = 8.27); and three for *LWS* on LG14 (LOD = 6.46), LG10 (LOD = 15.44) and LG16 (LOD = 4.18) (Figs. 1 and 3). All of these QTL are in *trans* to the double cone opsin genes that are located in two separate tandem arrays on LG5. Similar to the single cones, the normalization procedure leads to some degree of autocorrelations between the expression of the double cone opsins. As a result, we obtain overlapping QTLs for all the double cone opsins on LG16, and for *RH2A* and *LWS* on LG10 (Figs. 1 and 3). It is important to note that *SWS1, SWS2B* and *LWS* opsins have one overlapping QTL on LG14 that is independent of the normalization, suggesting that genetic cross-talk exists between the single and double cones (Fig. 1).

The LOD scores, QTL intervals and percent variance in opsin expression (PVE) explained by each of the QTLs are provided in Table 2. The double cone opsin QTL on LG16 explain 13.56, 18.2 and 6.67% of the F_2_ variance in *RH2B*, *RH2A* and *LWS* expression respectively. While the *LWS* QTL on LG10 is a major contributor accounting for 29.25% of the variance in the F_2_ expression, the overlapping *RH2A* QTL only explains 13.61% of its variation. Lastly, 10.65% variance in LWS expression is proportioned by the QTL on LG14.

**Table 2:** Summary of QTL for double cone opsin expression in the MA cross

Opsin	LG	Location (cM)	LOD	PVE	95% BI (cM)	95% BI (markers)
*RH2B*	16	64.19	4.97	13.56	51.27–71.89	RAD_10047 to RAD_19271
*RH2A*	16	67.5	8.27	18.2	51.27–71.89	RAD_10047 to RAD_19271
*RH2A*	10	54	6.37	13.61	42.9–54.86	RAD_14019 to RAD_39775
*LWS*	10	52.91	15.44	29.25	48.02–54.63	RAD_45540 to RAD_1385
*LWS*	14	52.5	6.46	10.65	44.97–56.81	RAD_41335 to RAD_49138
*LWS*	16	69	4.18	6.66	51.27–71.89	RAD_10047 to RAD_19271

*LOD* Logarithm of Odds, *PVE* Percentage of variance explained in the F_2_, *BI* 95% Bayes interval

Similar to the single cone opsin QTL on LG20, *RH2B* QTL on LG16 shows dominance, with equal levels of expression in F_2_ heterozygotes and *A. baenschi* homozygotes (MM<MA=AA) (Additional file 3: Figure S3A). While the expression of *RH2A* opsin on LG10 is additive (MM<MA<AA), heterozygous genotypes at LG16 have lower levels of *RH2A* expression than either of the *M. mbenji* or *A. baenschi* homozygotes, indicating underdominance (MM>MA<AA) (Additional file 3: Figure S3B). Lastly, the three loci regulating *LWS* expression on LG14, LG10 and LG16 demonstrate underdominance (MM>MA<AA), additivity (MM>MA>AA) and overdominance (MM<MA>AA) respectively (Additional file 3: Figure S3C).

### Candidate genes for opsin expression

The QTL intervals on LGs 14, 20, 17, 16 and 10 each map to distinct regions of the *O. niloticus* genome, spanning on average 8 Mb (The *O. niloticus* genome was used since the gene annotations were complete). Except for the *cis*-QTL on LG17, all the other *trans*-QTL contain numerous genes that potentially could contribute to the differential expression of the opsins. We have flagged certain genes as the most likely candidates based on their previous implication with opsin expression in the TA cross and/or association with photoreceptor function in other organisms (Table 3).

**Table 3:** Putative candidate genes for the differential expression of opsins in all QTLs except LG17

Gene	Location	Accession number	Function	Reference (if available)
*Paired box protein Pax3B*	LG14:3942202	XM_005461712.1	Involved in neural development.	[102]
*Zinc finger protein neurod4*	LG14:4686207	XM_005454306.1	Regulates the proliferation of photoreceptor progenitors	[81]
*Max-binding protein MNT*	LG14: 14694887	XM_005463727.1	Transcriptional repressor associated with retinitis pigmentosa.	
*T-box transcription factor TBX2b*	LG10:5759829	XM_005471922.1	UV photoreceptor specification in zebrafish	[27]
*Retinal guanylyl cyclase 2*	LG10:8655640	XM_003456801.2	Expressed with the cone cells in the retinas of zebrafish	[103]
*GPCR Kinase 1*	LG10:8655640	XM_003456840.2	Phosphorylates rhodopsin and leads to its deactivation	[82]
*Nuclear Receptor Subfamily 1D1*	LG20:25027615	XM_005478044.1	Interacts with *NR2E3* and plays critical role in photoreceptor development	[83]

For genes without references, all information was obtained from Genecards (http://www.genecards.org/)

### *SWS1* coding sequence comparison between *M. mbenji* and *A. baenschi*

**Fig. 4:**
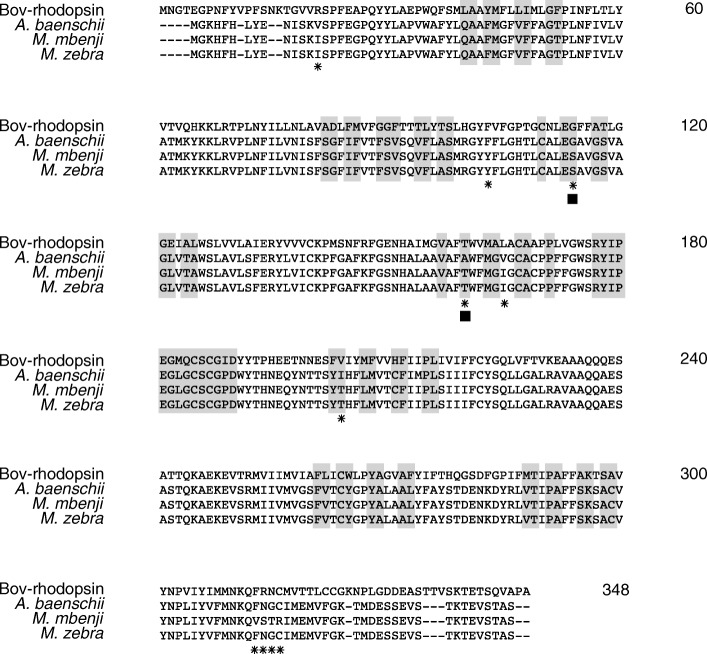
Comparison of coding sequences between *A. baenschi* and *M. mbenji.* Alignment of coding sequence of *SWS1* opsin from the two F_0_ of the cross, *M. zebra* and Bovine rhodopsin. Gray bars indicate amino acid residues present in the retinal binding pocket that are predicted to be spectrally tuning. 10 amino acids (indicated by asterisks) are different between *M. mbenji* and *A. baenschi*. 2/10 sites (indicated by the black square) represent substitutions that cause a change in the physical properties of the site. These two sites could alter the sensitivity of the *SWS1* opsin in *A. baenschi* relative to *M. mbenji*. Amino acids are numbered according to bovine rhodopsin

In both grandparents of the MA cross, *M. mbenji* and *A. baenschi*, the 5 exons of the *SWS1* gene encode a 336-amino acid opsin protein that shows extensive sequence similarity to the *SWS1* encoded by *M. zebra* (Fig. 4). However, the opsins differ from each other at 10 amino acid sites. We determined that 2 out of these 10 sites (corresponding to bovine rhodopsin sites 114 and 160) could potentially change the polarity of the amino acid and likely be spectrally tuning, thereby resulting in subtle distinction in the spectral sensitivity of the *SWS1* opsin between the two species.

### *Cis*-regulatory elements important for *SWS1* expression

**Fig. 5:**
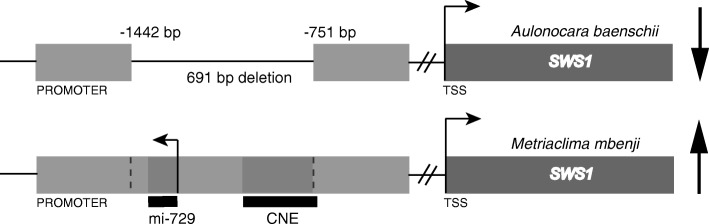
Cis-regulatory mutation in the *SWS1* opsin regulates its expression. Schematic representation of the promoter of the *SWS1* gene in both the F_0_ of the cross. Sequencing revealed a 691 bp deletion located 751 bp upstream of the translation start site (TSS) of the opsin in *A. baenschi* (low *SWS1* expression) that is absent in the promoter of *M. mbenji* (high *SWS1* expression). The region spanning the deletion comprises a miRNA *miR-729* (transcribed on the strand opposite to that of the opsin) and a conserved non-coding element (CNE). Expression of the *SWS1* opsin in both the species is denoted by the black arrow located to the right of the gene

To test for causative regulatory mutations for the differential expression of *SWS1* opsin, we sequenced approximately 2 kb of the promoter of the gene in both *M. mbenji* and *A. baenschi* F_0_ using overlapping primer sets. We found a 691 bp deletion located 751 bp upstream of the start codon of the *SWS1* gene in *A. baenschi* (low *SWS1* expression) that was absent in the promoter of *M. mbenji* (high *SWS1* expression) (Fig. 5). To test if this region was variable in other cichlid species, we sequenced 17 species from Lake Malawi with previously characterized opsin profiles. Although nine of the 17-species panel had low/negligible levels of *SWS1* expression, none of the species in the panel had the deletion in the *SWS1* promoter. We further analyzed this region for the presence/absence of the deletion in 29 additional species from Lake Malawi by size using gel-electrophoresis. Only one of the 29 species tested, *Placidochromis milomi,* shared the promoter deletion. Additionally, examination of cichlid genomes sequenced by Malinsky et al. [65] revealed that the same deletion was present in three species- *Aulonocara stuartgranti*, *Placidochromis milomi* and *Trematocranus placodon*. All three of these species have no/low *SWS1* expression. While the *Trematocranus* individual from our lab did not have the deletion, the sample from Malinsky et al. [65] did, suggesting within-species variation. This kind of individual variation is not unexpected and has been previously documented in Malawi cichlids [65]. Finally, neither of the representative cichlid species from Lake Tanganyika and Lake Victoria nor the two riverine ancestors had the deletion. All sampled species along with their opsin expression profiles are provided in Additional file 5.

**Fig. 6:**
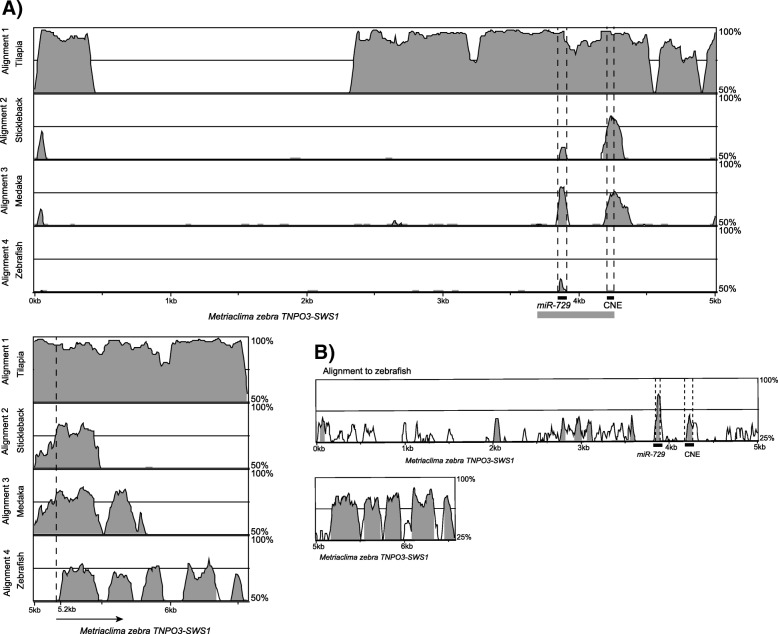
Phylogenetic footprinting of the *SWS1* promoter in teleosts. **a** VISTA alignment of the *SWS1* promoter region from tilapia, stickleback, medaka and zebrafish relative to *M. zebra* (X-axis) show that both the *miR-729* and CNE are conserved among teleosts. Grey peaks indicate more than 50% conservation in a 50 bp sliding windows; the arrow at the bottom indicates the start site of *SWS1* gene. Horizontal grey bar below the X-axis shows the location of the putative deletion in a few species from Lake Malawi. **b** VISTA comparison of the same region between *M. zebra* and zebrafish. Grey peaks indicate more than 25% sequence conservation in 50 bp sliding windows. Both the *miR-729* and CNE are marked by black bars below the horizontal axis

We used mVISTA to perform phylogenetic footprinting of the *SWS1* promoter of *M. zebra* (lacking the deletion) with orthologous regions from several other teleosts (tilapia, stickleback, medaka), including zebrafish. Our results show that the promoter of the *SWS1* gene bears a high degree of conservation for two well described elements- a miRNA (encoded on the strand opposite to that of the *SWS1* opsin) and a conserved non-coding element (CNE) (Fig. 6), both of which are located within the region spanning the deletion [66, 67].

Using mirBase, we found that the miRNA bears a high level of conservation with annotated zebrafish miRNA *dre-miR-729* (See Additional file 6). It is conserved across teleosts and work in medaka has shown that it is coexpressed with *SWS1* opsin in the same photoreceptors and that both the miRNA and *SWS1* opsin are bidirectionally transcribed using the CNE element [67].

We used the 3′ UTRs of all genes in the Tilapia genome (O_niloticus_UMD1, GCA_001858045.2), to find prospective targets for *miR-729*. Out of the ~ 28,000 genes predicted in the cichlid genome, we found that the *miR-729* could potentially interact with 1024 genes. Subsequent filtering of this list based on conserved roles in photoreceptor development and regulation enabled us to identify six top candidate genes that could play key roles in modulating opsin expression through their regulation by *miR-729* (Table 4). Similarly, in silico analysis of the CNE predicted several putative binding sites for transcription factors connected with photoreceptor development in other systems (Table 4).

**Table 4:** Potential targets of *miR-729* and transcription factors (TFs) with putative binding sites in the CNE

Putative targets of *miR-729*	Potential binding TFs in the CNE
1. *Retinoic acid receptor alpha-A* 2. *Retinoic acid receptor RXR-gamma-B* 3. *Cone-rod homeobox* 4. *Thyroid hormone receptor alpha* 5. *Homeobox protein SIX2* 6. *Thyroid hormone receptor-associated protein 3*	1. *Cone-rod homeobox* 2. *Neural Leucine Zipper* 3. *Orthodenticle Homeobox* 4. *Microphthalmia-Associated Transcription Factor* 5. *Retina and Anterior Neural Fold Homeobox* 6. *Visual System Homeobox* 7. *Ventral Anterior Homeobox*

## Discussion

In order to unravel the molecular mechanisms underlying the visual diversity in Lake Malawi cichlids, we utilized an intergeneric cross (*M. mbenji* X *A. baenschi*) and reduced representation sequencing of the F_2_ genome to find loci associated with cone opsin expression. We find a total of 12 QTL linked to the expression of six cone opsins between the two species, corresponding to five unique genomic loci. It is possible that the modest size of our F_2_ population restricted our power to detect additional small QTLs with minor effects and also might have led to an overestimation of the variances explained by the individual QTL [68]. Although the absolute variances for each QTL might be inflated, we hypothesize that the relative magnitudes of the identified QTL for each opsin are most likely accurate. Future fine-mapping with additional F_2_ individuals will help provide more exact estimates of the absolute variances explained by each QTL. Since our RAD-Seq approach for detecting QTLs relies on finding alternately fixed SNPs between the grandparents of the cross, it is also plausible that additional QTL might not have been identified.

### Regulation of *SWS1* opsin in Lake Malawi cichlids

QTL mapping revealed three QTLs associated with the differential expression of *SWS1*- two in *trans* on LG14 and LG20, and one located in *cis* to the *SWS1* opsin gene on LG17. The *cis*-QTL is the largest of the three QTL spanning a 7 Mb region on LG17 with the *SWS1* opsin gene located 20 bp from the most significant marker. Finding a QTL located in *cis* to the *SWS1* opsin is very surprising because results from this study as well as our previous TA cross suggest that all other opsins are regulated by multiple *trans* acting loci [62].

Comparison of the *SWS1* protein sequences revealed two amino acid differences between the parental species of the MA cross that could potentially alter the maximal sensitivity of the opsin. Based on evidence from previous site-directed mutagenesis analysis and microspectrophotometry (MSP) studies, we theorize that the shift would be very small, altering the sensitivity of the opsin by just a few (~ 10) nanometers [50, 69]. However, this inference is not without caveats and exact sensitivity of *SWS1* opsin in both parental species can only be determined by experimental verification [70]. Regardless, it is safe to say that the sensitivity of the visual system would be affected negligibly by the coding sequence changes, especially when compared to the substantial shift due to the change in opsin expression from *SWS1* to *SWS2B* (~ 65 nm) [52]. While adult *A. baenschi* have no appreciable expression of *SWS1* in their retinas, *M. mbenji* show high levels of expression [63]. We postulate that the differential expression of *SWS1* opsin is partly due to the *cis*-regulatory mutation in the *SWS1* gene. Specifically, the ~ 700 bp deletion located in the proximal promoter of *A. baenschi* might lead to the loss of putative enhancers needed to drive *SWS1* expression.

Our findings indicate that the region spanning the deletion in the *SWS1* promoter comprise a miRNA *miR-729* and a non-coding element (CNE) that is highly conserved in nearly 250 million years of teleost evolution. Such extensive conservation points to the functional importance of both elements to *SWS1* expression. Exactly how the *miR-729* and CNE modulate the *SWS1* gene and its expression in cichlids is still unclear. miRNA’s usually bind to the 3′ UTR of genes and work as post-transcriptional repressors [71]. It is possible that the *miR-729* prevents the misexpression of genes in the wrong cone type. For example, experimental work in mice has shown that *Retinoic Acid Receptor Gamma* (*RXR*γ) suppresses S-cone opsin (homologous to *SWS1* in teleosts) [72]. *RXR*γ is one of the putative targets of *miR-729* in cichlids. We hypothesize that in *M. mbenji* (lacking the deletion), *miR-729* works to keep the levels of *RXR*γ low, so that the *SWS1* cone opsin expression is not repressed. In *A. baenschi*, absence of the *miR-729* due to the deletion can lead to high expression of *RXR*γ, thereby preventing *SWS1* expression. Some of the putative transcription factors that can bind to the CNE in cichlids include upstream genes well-known to be involved with photoreceptor development such as *Cone Rod Homeobox* (*CRX*) and *Orthodenticle Homeobox 2* (*OTX2*). With *A. baenschi* missing these transcription factor binding sites, we postulate that its *SWS1* expression will be low.

It is interesting to note that some of the predicted targets for *miR-729* are the same transcription factors with putative binding sites in the CNE (Table 4). All cone cells arise from the same pool of retinal progenitor cells and it has been shown that the adoption of a specific cone fate depends on the precisely timed, coordinated and often repeated expression of key transcription factors [73, 74]. It is possible that the spatial and temporal expression of miRNA could act as a negative feedback loop and determine a certain cell-fate decision. Previous work in medaka has shown that the homologous *SWS1* CNE drives the coexpression of both the *miR-729* and the *SWS1* opsin in the same cone cells [67]. This *SWS1* cone-specific expression of *miR-729* in cichlids will be tested in the future with the help of reporter constructs. In order to conclusively prove that the *miR-729* and CNE play a role in moderating *SWS1* expression, future studies with CRISPR targets against the *miR-729* and specific sites within the CNE need to be carried out.

In trying to determine whether the *SWS1* promoter deletion was common, we found that only 4 of 50 species tested in Lake Malawi had the mutation. All species that had the deletion have low *SWS1* levels but all species with low *SWS1* expression did not have the deletion. This is not surprising because we found two other QTL that influence *SWS1* opsin expression in our cross. It is possible that the differential expression of *SWS1* is achieved by other mechanisms that arose in parallel in various species in Lake Malawi. Interestingly, species with the deletion were quite disparate and include two species of *Aulonocara,* one species of *Placidochromis,* and one species of *Trematocranus.* This suggests random sorting of the deletion into different lineages as occurs for ancestral polymorphisms. None of the species outside the lake, particularly species from Lake Victoria (*P. nyererei*) and Tanganyika (*N. brichardi*) and the riverine species (*O. niloticus* and *A. burtoni*) had the deletion, indicating that the deletion in the *SWS1* promoter likely arose within Lake Malawi and is being shared either by hybridization or sorting through the flock as standing genetic variation [37, 75]. Future work looking at whole genomes from various species in each of these lakes will enable us to identify if other species carry the deletion.

### Opsin expression in cichlids is controlled by multiple, overlapping QTL

With the exception of one *cis*-QTL for *SWS1* on LG17, our results indicate that most opsins in cichlids are regulated by *trans* loci located in genomic regions distant from the opsins. Being upstream of the immediate genes regulating the trait, *trans* changes are often thought to be inefficient because of the widespread and often negative pleiotropic effects achieved by simultaneously affecting multiple downstream targets [76]. We speculate that in cichlids, *trans* mutations might actually be favored for two main reasons. First, evidence from other organisms points to the existence of Locus Control Regions (LCRs) that modulate the expression of downstream opsin tandem arrays [77]. These LCRs are usually driven by multiple *trans* acting factors operating as switches. By virtue of its sequestered location (LG17), *SWS1* opsin need not be driven by a LCR and thus its promoter might be susceptible to accumulating *cis*-changes. Second, closely related species in Lake Malawi often exhibit coordinated differences in the expression of multiple opsins [78]. Such synchronized changes can be more easily achieved by a single *trans* change influencing multiple downstream opsins as opposed to individual *cis* mutations in each of the opsin promoters.

The QTL for single cone (LG14, LG20 and LG17) and double cone opsins (LG10 and LG16) are co-localized to the same chromosomal regions. Although this might be a direct outcome of the normalization procedure used in the study, previous in-situ hybridization work both in the hybrids of the MA cross as well other Malawi species have shown that in conditions of altered light spectra, both the single cone and double cone opsins are co-expressed in their respective cell types in certain parts of the retina [63, 79, 80]. Therefore, it is not unreasonable to hypothesize that at least a few loci controlling their expression might be shared as well. Co-mapping of a QTL for *LWS* with 2 QTLs for *SWS1* and *SWS2B* opsins, independent of the normalization, is striking. Such genetic coupling between the single and double cone opsins hints at pleiotropic effects, although at this stage multiple linked genes cannot be ruled out. Future fine-mapping of the shared QTL interval on LG14 will help resolve this issue.

Previously, Castle-Wright estimates based on gene expression levels in the three generations of the cross (F_0_, F_1_ and F_2_) suggested that ~ 8 loci govern differences between *SWS1/SWS2B* opsin expression in the MA cross [63]. In the current study, we were able to detect only 3 of 8 loci for these opsins. Failure to recognize additional loci might be due to the complex architecture of the single cone opsins including multiple genes with pleiotropic and epistatic effects along with dominance. For the green-sensitive opsins in the double cones (*RH2A* and *RH2B*), we were successful in identifying 3 QTL on LG10 and LG16. This was more than what had been previously predicted from the Castle-Wright estimation. The *LWS* opsin exhibits transgressive expression in the F_2_ generation of this hybrid cross, which we previously theorized was due to the presence of two loci with opposing effects in each parent [63]. Subsequent segregation of the alleles with effects in the same direction leads to F_2_ offspring with high expression levels. Consistent with our hypothesis, in the present study we found 3 QTL for *LWS* opsin with divergent effects. While the inheritance of *M. mbenji* alleles in the largest QTL on LG10 increases *LWS* expression, the *M. mbenji* alleles seem to decrease expression in the other QTL on LG14 and LG16. Failure to find the fourth QTL for *LWS* as projected by the Castle-Wright estimator might be due to the limitations discussed above.

Together with our previously published QTL generated from the TA cross, we now have a total of 23 QTL linked to the differential expression of all six cichlid cone opsins in cichlids. Of the 23 QTL, two (*RH2A* and *LWS* on LG10) replicate between the two studies. Also, the putative QTL for *SWS2A* expression on LG23 (which we discarded in this study, for reasons- see Results) matches the QTL of the same opsin from the TA cross. The common parental species between the two crosses, *A. baenschi* might be the source of these replicate QTL. Nevertheless, we postulate that QTL results from both the crosses can be combined for two main reasons. First, different species in Lake Malawi all diverged from a recent ancestor less than a million years old [41]. This recent evolutionary history resulted in the maintenance of a high degree of similarity between the genomes of different species. Most cichlid species have conserved genomic structure, with contiguous stretches of nearly identical sequence. Second, standing genetic variation, mostly as ancestrally-inherited polymorphisms, seems to be an important source for genetic diversity in cichlids [75]. Repeated selection of the same allele independently by different species can lead to shared genetic mechanisms. Currently, fine-mapping and identification of candidate gene (and causative mutation) in the QTLs from the TA cross is in progress in the lab. It will be interesting to see if the same genes can explain the variation in opsin expression in the MA cross as well.

### Candidate genes for differential expression of opsins

All the *trans* QTL identified in this study correspond to discrete regions in the genome and contain numerous candidate genes. Although the list provided in Table 3 is drastically condensed, it is the first step towards identifying suitable candidates and also provides a point of reference for our fine-mapping efforts. There is the possibility that the differential expression of the opsins in these regions is being regulated by other factors such as microRNAs, non-coding RNAs and epigenetic elements that we have not considered here.

One of the candidate genes for *LWS* expression is *T-box Transcription Factor TBX2B*, located in the QTL on LG10. In zebrafish, this gene has been implicated in promoting the production of *SWS1* cone cells by repressing the rod differentiation pathway [27]. Another good candidate is *NEUROD4*, a cell cycle regulator gene on LG14 that promotes photoreceptor fate, while inhibiting other retinal cell fates in zebrafish [81]. Other genes connected to photoreceptor function thereby making them suitable candidates include *GRK1* on LG10 (Phosphorylates the rod opsin leading to its inactivation) [82], and *NR1D1* on LG20 (Affects the survival of the S-cones; mutations in this gene lead to increased sensitivity to blue light in humans) [83].

## Conclusions

In this study, we have shown that both *cis* and *trans* regulatory changes are associated with the divergence in photoreceptor sensitivity in African cichlids. Additionally, we uncover a regulatory deletion in close proximity to the start site of the *SWS1* opsin that likely aids its expression variation in these fishes. This is our second regulatory deletion that we have identified as possibly playing a role in cichlid opsin expression (see [84]) suggesting that indels may be an important source of variation and might contribute to cichlid explosive radiation.

Expression of *SWS1* opsin in Lake Malawi cichlids is tied to their visual ecology, having been previously correlated with foraging and predatory behavior [53, 85]. Zooplanktivorous cichlids and algal browsers in Lake Malawi usually have higher *SWS1* expression than species that do not rely on plankton for their nourishment. Furthermore, behavioral evidence in cichlids with *SWS1* expression shows that the duration of finding prey is significantly increased under illumination conditions devoid of ultraviolet input. Finding the potential *cis*-regulatory deletion and other loci associated with its differential expression could thus serve as the first step towards understanding the adaptive origins of this trait.

## Methods

### Genetic cross

F_2_ hybrids generated from an intergeneric cross between two Lake Malawi species *Aulonocara baenschi* and *Metriaclima mbenji* (MA cross) were sampled for this study [63]. Briefly, hybrid progeny were generated from a mating between a single *A. baenschi* male and a single *M. mbenji* female. *M. mbenji* parental specimen used for the cross was third generation lab-raised progeny generated from a wild-caught stock maintained in the aquaculture facility at the University of Maryland. The *A. baenschi* parent was multiple generation progeny generated from fish obtained from the aquarium trade. A single male and ten females from the F_1_ generation were randomly chosen and then mated to generate 350 F_2_ progeny (~ 40 F_2_ families). All individuals used in this study were sexually mature (> 6 months of age) and raised under laboratory conditions. Sampling was done randomly with respect to sex. Following euthanasia with a lethal dose of buffered MS-222, retinas were dissected and stored in RNAlater at -20C.

### Phenotyping opsin expression

Opsin expression in the F_2_ hybrids was quantified as described previously [63]. RNA was extracted from dissected retinas and transcribed into cDNA with Invitrogen Superscript III (Invitrogen, NY, USA) following manufacturer instructions. Relative opsin expression from each cDNA sample was measured by real-time, quantitative PCR (RT-PCR) using opsin specific Taqman primers and probes on a Roche Lightcycler 480 according to previously published protocols [47, 86, 87] (*RH2Aα* and *RH2Aβ* genes that are more than 99% identical were combined and noted as *RH2A*). Gene expression was normalized as a percent of total opsin expression within single cones and double cones respectively. Therefore, the fraction, *f*, of *SWS1* expression in the single cones is calculated as:

f_*SWS1*, SC_ = T_*SWS1*_/(T_*SWS1*_ + T_*SWS2B*_ + T_*SWS2A*_)

where T is the transcript level for each of the single cone opsin genes. Similarly, the fraction of *LWS* opsin expression in double cones is:

f_*LWS*, DC_ = T_*LWS*_/(T_*RH2A*_ + T_*RH2B*_ + T_*LWS*_)

### RAD-Seq libraries

To identify SNPs for QTL mapping, restriction-site associated DNA sequencing (RAD-Seq) was performed on F_2_ progeny. Reduced representation libraries were constructed by Floragenex Inc. (Portland, OR, USA) according to the protocol of Baird et al. [88]. High-molecular weight genomic DNA was extracted from fin clips of 157 F_2_ hybrids and the 2 F_0_ parents using DNeasy blood and tissue kits (Qiagen Inc. CA, USA) and quantified on a BioTek FLX800 fluorometer (BioTek, VT, USA) with Quant-iT dsDNA High-sensitivity kit (Invitrogen, NY, USA). 20 ng/ul DNA from each sample was digested with HF-SbfI restriction enzyme, ligated to 95 unique barcode linkers and combined into two libraries (the second library had 66 F_2_ and the two F_0_ individuals). Each library was then randomly sheared to an average size of 500 bp and size selected between 300 and 500 bp. Following ligation with Illumina adapters and PCR enrichment, both libraries were gel purified and quantified, before being sequenced single-end on an Illumina Hi-Seq 2000 at the University of Oregon Genomics Core Facility.

Raw sequence reads were filtered for quality using the FASTX toolkit. In silico analysis for identification of SNPs and determination of genotypes was performed with the built-in wrapper program *denovo_map.pl* in Stacks (version 1.35) as described by Catchen et al. [89]. Briefly, demultiplexed reads of each individual were assembled into unique loci and heterozygous alleles were identified (allowing for a maximum of 1 mismatch). Then, orthologous stacks from the F_0_ parents were matched into a common catalog of loci and differentially fixed SNPs were identified between them. Lastly, the F_2_ stacks were compared to the parental stack catalog to infer genotypes at each locus. Conservative criteria were imposed for inferring genotypes from stack loci. Default parameters were modified to require the minimum number of reads to call homozygous genotype in the F_2_ to be 10 (*min_hom_seqs*), the minor allele frequency below which a stack is deemed a homozygote to be 1/15 or 0.066 (*min_het_seqs*); the minimum frequency of minor allele to be called a heterozygote to be 2/15 or 0.133 (*max_het_seqs*). Genotypes with minor allele depth between 0.066–0.133 were considered to not have adequate support and were deemed ‘unknown’. The RAD loci were filtered further to exclude markers that were not differentially fixed between the F_0_ parents. Finally, the consensus sequence of each RAD marker was aligned to the M_zebra_UMD1_genome assembly using BLAST [90]. Only markers with unique hits in the *M. zebra* genome were included for consequent analyses.

### Linkage map construction

Genetic linkage mapping was performed in R/qtl (R Foundation for Statistical Computing) following the guidelines described in Broman and Sen [91]. Markers were excluded from subsequent analyses if they were not genotyped in at least 90% of the individuals or failed to satisfy Hardy-Weinberg expectations (after correcting for multiple comparisons). All the RAD-Seq markers were grouped into linkage groups by requiring a maximum recombination frequency of 0.35 and a minimum log_10_ odds ratio (LOD) threshold of 10. Within each linkage group, markers were then ordered by repeatedly rippling the order of a 7-marker sliding window, ultimately choosing the order with the fewest obligate crossovers. Large-scale changes in marker order were made manually and markers that did not fall into linkage groups were dropped from the analysis. A LOD score for each genotype was calculated in order to identify statistically unlikely events, such as closely placed double crossovers. After excluding such incorrect genotypes, final intermarker distances were estimated using the Kosambi map function [92]. Lastly, linkage groups (LGs) in the constructed map were aligned to the anchored *O. niloticus* (O_niloticus_UMD1) genome assembly to assign to the correct cichlid LG nomenclature [93].

### QTL analyses

Quantitative trait loci (QTL) regulating opsin expression were detected following methods in O’Quin, et al. [62]. QTL analyses were performed in R/qtl using the fully automated model selection algorithm *Stepwise* that searches for multiple interacting QTL for each opsin [91]. With the stepwise analysis, LOD of association between opsin expression and the genotypes at every marker were computed using forward selection to a model with 10 QTL, followed by backward elimination to the null model of no QTL. Missing genotype data at the putative QTL were taken into account by using Haley Knot regression. The function *fitqtl* was used to calculate the LOD score and percentage of variance in opsin expression explained by each QTL. Significance thresholds for the QTL were determined from the 95th percentile of the genome-wide LOD scores calculated from 1000 permutations. Markers with the highest LOD score were used to test the effect of each QTL. Interval estimates of the location of the QTL were obtained from 95% Bayes credible intervals (BI) extended to the nearest genotyped markers. For each QTL, candidate genes were estimated by matching the markers flanking the Bayes intervals (BI) to their corresponding regions in the *O. niloticus* (O_niloticus_UMD1) genome [93].

### *SWS1* coding sequence analysis

*SWS1* coding sequences from *M. mbenji*, *A. baenschi* and *M. zebra* were obtained from Genbank (Accession numbers: *M. mbenji*: HM049223.1, *A. baenschi:* GQ422525.1, *M. zebra*: NM_001310074.1). Following translation using the Expasy Translate tool, amino acid sequences from all three species were aligned along with bovine rhodopsin (UniProtKB P02699) using *ClustalW (version 2)* to identify amino acid substitutions that might be functionally relevant [94]. Attention was paid to sites where the substitution altered amino acid polarity, specifically in the retinal binding pocket of the opsin protein, similar to previous methods [69].

### *SWS1* promoter sequencing

The promoter of the *SWS1* gene was Sanger sequenced with overlapping primer sets in both the F_0_ of the cross as well as in a panel of 17 species from Lake Malawi with previously characterized opsin expression profiles. Retinas of the 17-species panel were collected during a field expedition to southern Lake Malawi in 2008 [78]. The primers were designed based on *M. zebra* genome (Primer sequences provided in Additional file 3: Table S1). DNA from each sample was PCR amplified, size selected by gel-electrophoresis, and sequenced using Applied Biosystems Big Dye Terminators on a ABI 3730xl. For each species, sequences from all the primers were assembled and edited manually in Geneious (*version 11.0.5*) before making comparisons [95]. The *SWS1* promoter sequence alignment file for the two parental species of the cross and the 17-species panel has been submitted to Genbank (Accession numbers: MH122659, MH122660 and MH215441 to MH215457). Since sequencing showed that the deletion was identical across species, 29 additional Lake Malawi species (with known opsin expression) were genotyped for the presence or absence of the deletion by fragment size on an agarose gel [84]. Further, whole genomes from 14 species native to the Malawi catchment area, were obtained from the Wellcome Sanger Institute [65] (12 of the Wellcome genome species are also included in the 29 species Malawi panel, and 2 species- *Aulonocara stuartgranti* and *Astatotilapia calliptera* are not). Sequence data from each species was aligned to the *M. zebra* reference assembly before looking for differences in the *SWS1* promoter. Finally, previously published genomes of cichlids archetypal of Lake Tanganyika (*Neolamprologous brichardi*), Lake Victoria (*Pundamilia nyererei*) and two ancestral riverine species (*Astatotilapia burtoni* and *Oreochromis niloticus*) were also examined to look for the deletion in the *SWS1* promoter [42]. All sampled species along with their single cone opsin expression profiles are provided in Additional file 5.

### Phylogenetic footprinting assay

DNA spanning from the last exon of *TNPO3* (the gene upstream of *SWS1*) to the first few exons of the *SWS1* gene were obtained from the *M. zebra* genome assembly (LG17: 12796067–12,802,659). Orthologous DNA regions spanning the same interval were obtained from the genome assemblies of four other teleost fish- Tilapia (*Oreochromis niloticus*, Tilapia_Broad assembly (LG17: 11751000–11,756,599), medaka (*Oryzias latipes*, NIG/UT MEDAKA1/oryLat2 assembly, scaffold1021: 45422–53,836), stickleback (*Gasterosteus aculeatus*, Broad/gasAcu1 assembly, chrUN: 22268595–22,274,962) and zebrafish (*Danio rerio*, GRCz10/danRer10 assembly, chr4: 13578911–13,588,818). Comparative sequence analysis was implemented in mVista (accessible at http://www-gsd.lbl.gov/vista/) to perform pairwise alignments of orthologous DNA sequences from multiple species [96]. Sequences were aligned with the global alignment tool MLAGAN and regions were deemed as conserved elements if they showed 50% identity within a 50 bp window for all species except zebrafish [97]. Since the divergence time between *M. zebra* and zebrafish is nearly twice as much (230 million years) as that of its next distant relative- stickleback (117 million years), the conservation parameters in zebrafish were relaxed and set to 25% identity within the same 50 bp interval (*M. zebra*- medaka: 98 million years, and *M. zebra*- Tilapia: 25 million years) [98].

Transcription factor binding site (TFBS) analysis on the CNE in the promoter of the *SWS1* opsin was performed using the JASPAR CORE Vertebrata 2016 database [99]. Settings were adjusted to shortlist putative transcription factor binding profiles with a relative score of ≥0.80. The identity of the microRNA (miRNA) was established using mirBase [100]. Potential target genes for the miRNA were predicted using the computational algorithm *miRanda* according to the methods described in Enright et al. [101]. 3′ UTR FASTA sequences for all cichlid genes were obtained from the Tilapia Broad assembly.

## Additional files

Additional file 1: RAD-Sequencing processing statistics for F_0_ and F_2_ in the cross. (XLSX 56 kb)

Additional file 2: FASTA sequences of the 1217 RAD markers for the construction of linkage map and QTL analysis. Their positions in the cichlid genome are also provided. (XLSX 125 kb)

Additional file 3: Figures for the linkage map used for QTL analysis and effect plots for single and double cone opsin QTL are provided. Also sequencing primers used for the promoter of *SWS1* opsin are also provided. (PDF 284 kb)

Additional file 4: Linkage map along with phenotype and genotype data used for the QTL analysis. Relative opsin expression (in percentage) of all F_2_ individuals used for the QTL study is provided. Opsin expressed is measured relative to total opsin expression within single cones and double cones respectively. The genotypes of all individuals at every marker are also denoted. Linkage positions of all markers used in the study are also provided. (XLSX 752 kb)

Additional file 5: All species tested for the presence or absence of deletion in the promoter of the *SWS1* opsin. This list includes the parental species of the cross, sampled species from Lake Malawi, and species from the Wellcome and Broad genomes. Opsin expression expressed as percentage of total opsin expression in single cones is provided for each species. (XLSX 49 kb)

Additional file 6: Alignment of the zebrafish *dre-mi-729* with teleost sequences in the region upstream of *SWS1* opsin. Highly conserved nucleotide positions are indicated in green. The predicted cichlid miRNA is highly conserved with zebrafish miRNA *dre-mi-729*. (PDF 262 kb)

## Abbreviations

BI: Bayes interval
CNE: Conserved non-coding element
LCR: Locus control region
LG: Linkage group
LOD: Logarithm of odds
MA cross: *Metriaclima mbenji* and *Aulonocara baenschi* cross
miRNA: microRNA
MSP: Microspectrophotometry
PVE: Percent variance in expression
QTL: Quantitative trait locus
RAD-Seq: Restriction-site associated DNA sequencing
SNP: Single-nucleotide polymorphism
TA cross: *Tramitochromis intermedius* and *Aulonocara baenschi* cross
TFBS: Transcription factor binding site
UTR: Untranslated region
