# Supplementary material for: Multiple trans QTL and one cis-regulatory deletion are associated with the differential expression of cone opsins in African cichlids
Source: BMC Genomics. 2018 Dec 18;19:945. doi: 10.1186/s12864-018-5328-z (PMC6299527; doi:10.1186/s12864-018-5328-z)
Supplement: Supplementary file 3 — Figures for the linkage map used for QTL analysis and effect plots for single and double cone opsin QTL are provided. Also sequencing primers used for the promoter of SWS1 opsin are also provided. (PDF 284 kb) [file 12864_2018_5328_MOESM3_ESM.pdf]

Additional File 3

Fig S1: Linkage map of the cross between *M. mbenji* and *A. baenschi*

Linkage map constructed from 157 F2 hybrids of an intergeneric cross between two species from Lake Malawi. The map includes 1217 markers in 22 linkage groups spanning 1558.1cM. RAD markers used to construct the map are labeled on the right side of each linkage group. For sites that have more than one marker, the number of additional markers at the location are labeled in brackets. Genetic distances in cM is depicted on the right. The linkage groups are named according to orthologous linkage groups in Tilapia.

Fig S2: Mean opsin expression among genotypic classes at each single cone QTL

Effect plots for each opsin at the marker with the highest LOD score in each QTL. In each plot, MM: *M. mbenji* homozygote, AA: *A. baenschi* homozygote, MA: heterozygote. Error bars indicate standard deviation.

A) *SWS1* expression

B) *SWS2B* expression

Fig S3: Mean double opsin expression among genotypic classes at each QTL

Effect plots for each opsin at the marker with the highest LOD score in each QTL. In each plot, MM: *M. mbenji* homozygote, AA: *A. baenschi* homozygote, MA: heterozygote. Error bars indicate standard deviation.

A) *RH2B* expression

B) *RH2A* expression

C) *LWS* expression

Supplementary Table 1: Sequencing primers for the promoter of the *SWS1* opsin gene.

Primer Name	Sequence 5’-3’
SWS1_promoter_3_F	ctggtccaggatgaaaatgg
SWS1_promoter_3_R	gccaaccagctcactgttgt
SWS1_promoter_2_R	ctcagagtccggagaacgtc
SWS1_promoter_1_F	tacctcgcttctcacccact
SWS1_mnpromoter_F	cctcaatgctccaaaaagga
SWS1_mnpromoter_R	cagacaggggccaggtaata

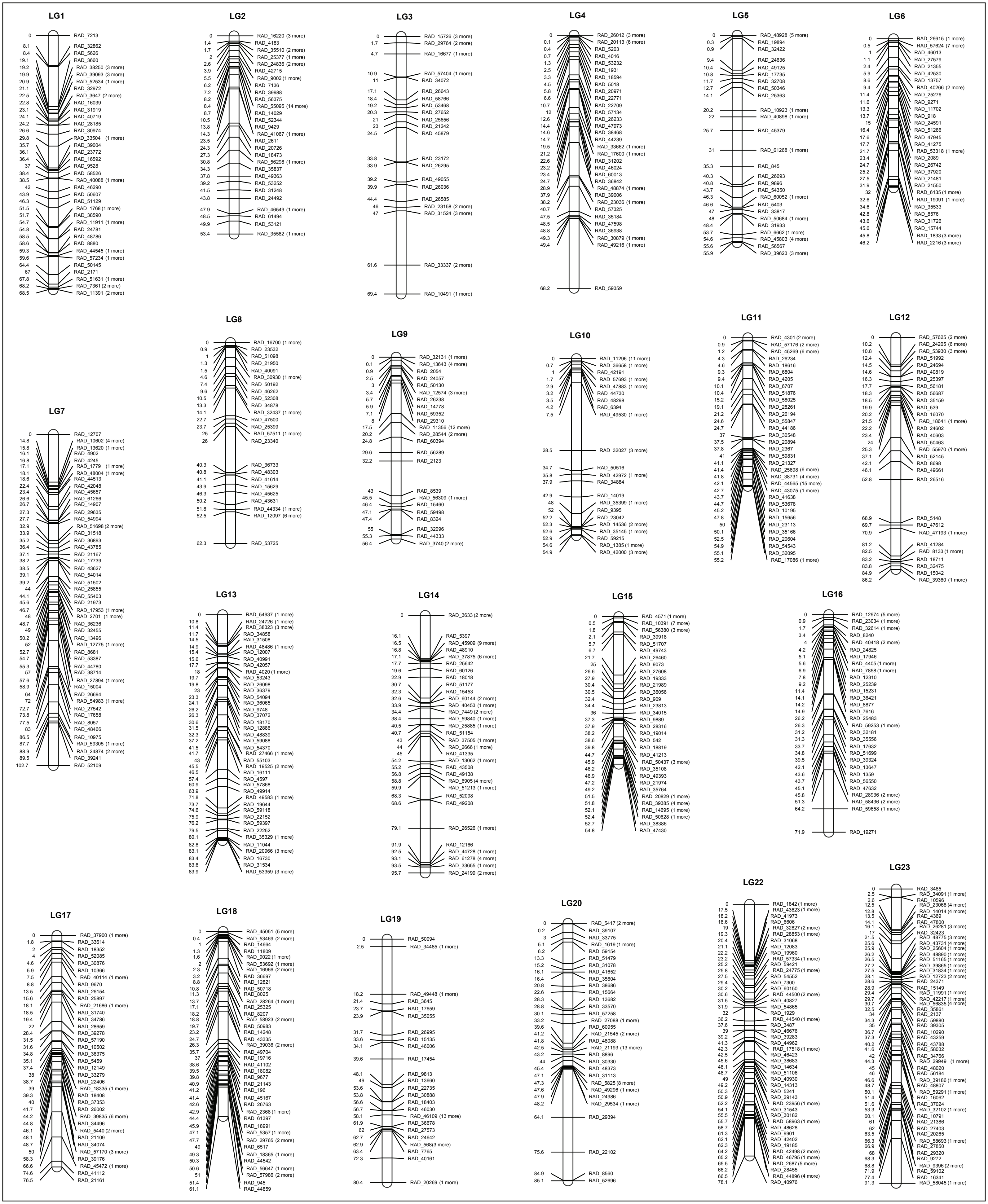
LG1
LG2
LG3
LG4
LG5
LG6
LG7
LG8
LG9
LG10
LG11
LG12
LG13
LG14
LG15
LG16
LG17
LG18
LG19
LG20
LG22
LG23

Fig. S2

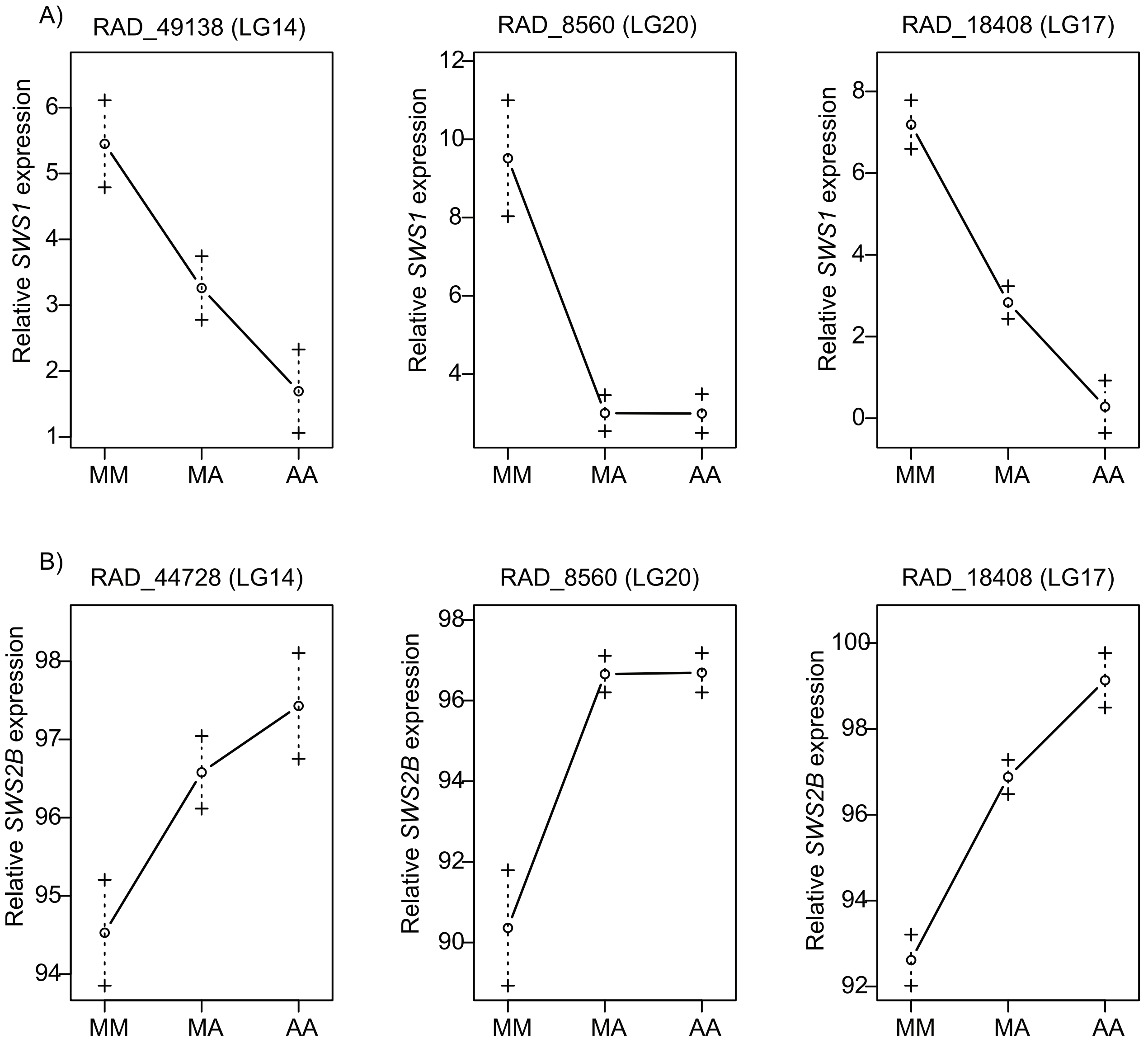
A)
RAD_49138 (LG14)
RAD_8560 (LG20)
RAD_18408 (LG17)
Relative SWS1 expression
MM
MA
AA
B)
RAD_44728 (LG14)
RAD_8560 (LG20)
RAD_18408 (LG17)
Relative SWS2B expression
MM
MA
AA

Fig S3

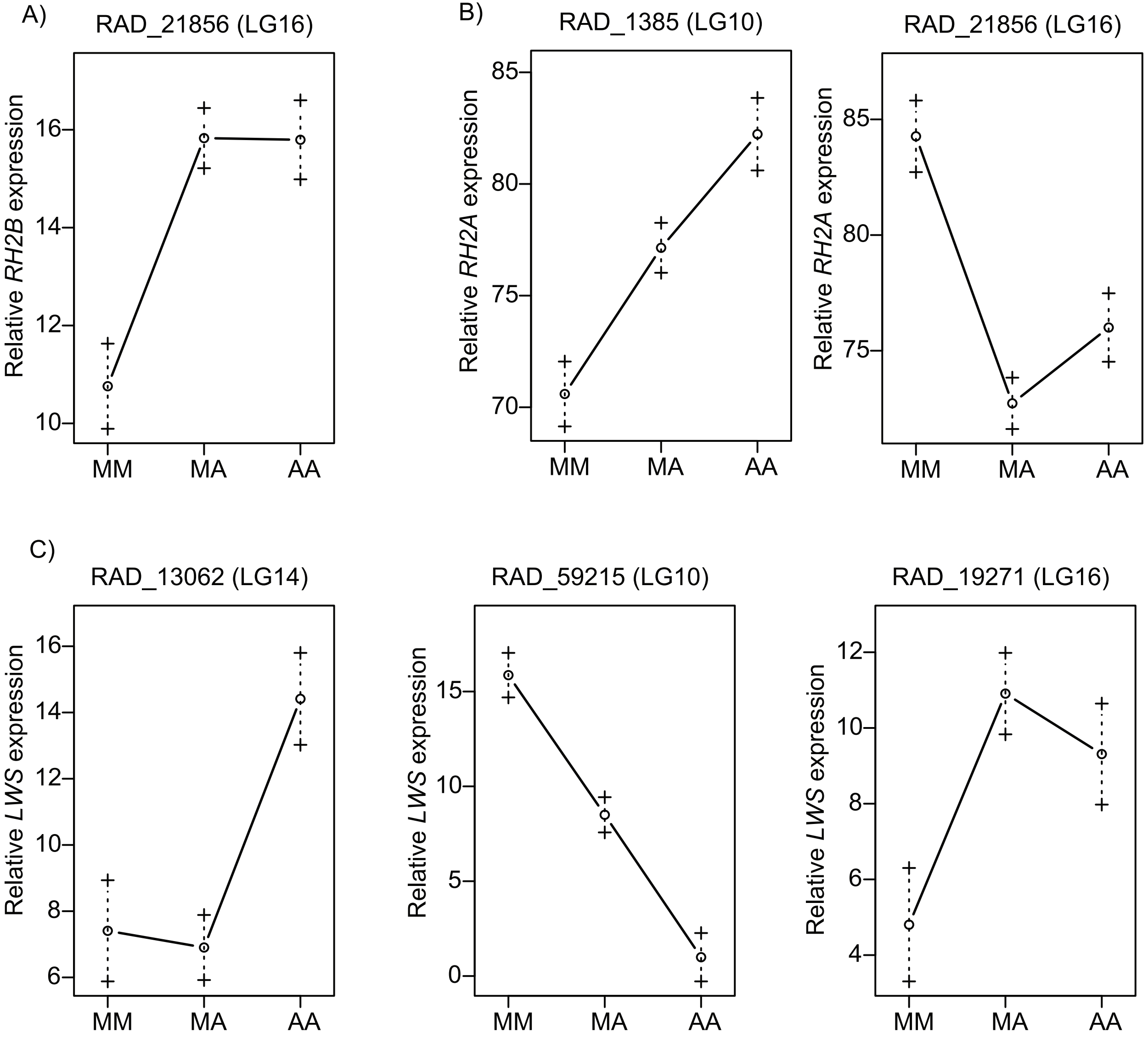

A)
RAD_21856 (LG16)
Relative RH2B expression
10
12
14
16
MM
MA
AA
B)
RAD_1385 (LG10)
Relative RH2A expression
70
75
80
85
MM
MA
AA
RAD_21856 (LG16)
Relative RH2A expression
75
80
85
MM
MA
AA
C)
RAD_13062 (LG14)
Relative LWS expression
6
8
10
12
14
16
MM
MA
AA
RAD_59215 (LG10)
Relative LWS expression
0
5
10
15
MM
MA
AA
RAD_19271 (LG16)
Relative LWS expression
4
6
8
10
12
MM
MA
AA
