# Supplementary material for: Multiple trans QTL and one cis-regulatory deletion are associated with the differential expression of cone opsins in African cichlids
Source: BMC Genomics. 2018 Dec 18;19:945. doi: 10.1186/s12864-018-5328-z (PMC6299527; doi:10.1186/s12864-018-5328-z)
Supplement: Supplementary file 6 — Alignment of the zebrafish dre-mi-729 with teleost sequences in the region upstream of SWS1 opsin. Highly conserved nucleotide positions are indicated in green. The predicted cichlid miRNA is highly conserved with zebrafish miRNA dre-mi-729. (PDF 262 kb) [file 12864_2018_5328_MOESM6_ESM.pdf]

```
> Medaka       ---------CTCCAAACTGAGGTCGTATCATACCCATGCTC-GTTCA-GCTCACAGCCTG
> Tilapia      ---------CTCCAAACTGAGGTCGTATCATACCCATGCTCCATGCA-GCTCACAGCCTG
> Stickleback  ------------CAAACTGAGGTCGTATCATACCCATGCTT-CTAAA-ATAACCAGACTG
> dre-mir-729  CATCACCATTCTGTAACCCAGGTCGTATCATACCCATGCATCATGGACACTTACAGCTTG
> Zebrafish    --------------AACCCAGGTCGTATCATACCCATGCATCATGGACACTTACAGCTTG

> Medaka       GTTATGATACAGCCCC-----------------------
> Tilapia      GTTATGATACAGCCCCCGCTCT-----------------
> Stickleback  GTTATGATACAGCCCCAGCTTT-----------------
> dre-mir-729  GTTACAATACAACCCCGGGTCACTGGATGGCCCTTGACG
> Zebrafish    GTTACAATACAACCCC-----------------------
```
